# Comparison of the Therapeutic Effect of Treatment with Antibiotics or Nutraceuticals on Clinical Activity and the Fecal Microbiome of Dogs with Acute Diarrhea

**DOI:** 10.3390/ani11061484

**Published:** 2021-05-21

**Authors:** Giulia Pignataro, Roberta Di Prinzio, Paolo Emidio Crisi, Benedetta Belà, Isa Fusaro, Carlo Trevisan, Luigi De Acetis, Alessandro Gramenzi

**Affiliations:** 1 Faculty of Veterinary Medicine, University of Teramo, 64100 Teramo, TE, Italy; rdiprinzio@unite.it (R.D.P.); bbela@unite.it (B.B.); ifusaro@unite.it (I.F.); agramenzi@unite.it (A.G.); 2 Veterinary Practice, “Ambulatorio Veterinario Dr. Carlo Trevisan”, 66016 Guardiagrele, CH, Italy; gledd@hotmail.it; 3 Veterinary Practice, “Ambulatorio Veterinario Dr. Luigi De Acetis”, 65023 Caramanico Terme, PE, Italy; luigi.deacetis@gmail.com

**Keywords:** dog, acute diarrhea, Canine Acute Diarrhea Severity Index, nutraceutical, antimicrobial resistance, antibiotic-sparing therapy, gut microbiota, Dysbiosis Index

## Abstract

**Simple Summary:**

Acute diarrhea in dogs is one of the most common reasons for veterinary visits. Although this disorder is generally self-limiting, antibiotics are still frequently used as treatment for acute diarrhea in clinical practice. Antimicrobial resistance represents a major challenge for public health and requires immediate and drastic solutions. To date, the emergence and spread of antimicrobial resistance has been attributed to the misuse or indiscriminate use of antibiotics. The aim of this study is to compare the effects on clinical activity and fecal microbiota of the administration of an antibiotic combination in comparison to a nutraceutical product in dogs with acute non-hemorrhagic diarrhea. The results of the present study suggest that this nutraceutical treatment had a similar clinical effect compared to the antibiotic formulation and may represent an alternative to commonly used antimicrobial therapy.

**Abstract:**

Dogs with acute diarrhea are often presented to clinical practice and, although this generally represents a self-limiting condition, antibiotics are still frequently used as treatment. The aim of this study was to evaluate the effects in dogs with acute non-hemorrhagic diarrhea of the administration of an antibiotic combination in comparison to a nutraceutical product. Thirty dogs were enrolled and randomly assigned to two groups: 15 dogs (group A) received a nutraceutical commercial product while 15 dogs (group B) received an antimicrobial combination of metronidazole and spiramycin. For each dog, the Canine Acute Diarrhea Severity Index, the fecal microbiota and the Dysbiosis Index were assessed. Both stool consistency and frequency decreased on day 2 in the dogs of group A compared to baseline, while in group B, these parameters significantly decreased at days 3 and 4. The global concern for rising antibiotic resistance associated with indiscriminate use of antimicrobials, in both humans and animals, suggests the necessity of avoiding empirical and injudicious use of these molecules in diarrheic dogs. These results suggest that the nutraceutical treatment had a similar clinical effect compared to the antibiotic formulation, representing a valid antibiotic-sparing therapeutic approach in canine acute diarrhea.

## 1. Introduction

Dogs with acute diarrhea are often presented to clinical practice. Common causes of acute diarrhea include inappropriate food intake, abrupt dietary changes, scavenger behavior and hypersensitivities. Alongside dietary causes, diarrhea can be secondary to adverse medication reactions and can have an infective origin with several pathogens associated with acute episodes in dogs [1,2]. However, non-complicated acute diarrhea often resolves spontaneously [3] and the exact cause is rarely determined in clinical practice.

Nevertheless, acute diarrhea may cause significant stress to both the pet and owner, and a rapid resolution of clinical signs is important in order to reassure the owner and to avoid any complications related to the persistence of diarrhea, such as dehydration and/or electrolyte imbalances. A range of therapeutic options are available, either targeting potential infectious agents and/or clinical signs. Antimicrobial prescription has been recorded in between 45% and 70% of canine diarrhea cases [4,5,6,7] and a recent case–control study [8] reported that in dogs with acute diarrhea, systemic antimicrobials were the most prescribed pharmaceutical agents, especially if hemorrhagic diarrhea or/and body temperature of more than 39.0 °C are present. These findings most likely reflect a perception that such clinical signs increase the likelihood that an infectious process is involved and/or that the intestinal mucosal integrity is lost, increasing the risk of bacteremia [9].

While antibiotics are still frequently used in the treatment of acute diarrhea in clinical practice, there are concerns about the use of antimicrobial agents due to their potential adverse effects on the balance of intestinal microbiota and as a potential cause of the spread of antibiotic resistance in animals [10].

Dogs with acute diarrhea had different microbial communities compared to healthy dogs and lower bacterial diversity was observed in regard to species richness [2,11].

This is one reason why interest in alternative interventions is increasing [12]. The important role of the intestinal gut microbiota in host health has resulted in a lot of interest in manipulating the composition of intestinal microbiota using probiotics and prebiotics.

Probiotics are defined as supplements or foods that contain viable microorganisms with a proven benefit to the host while prebiotics are supplements or foods (often dietary fibers or carbohydrates) that selectively stimulate the growth and/or activity of indigenous microorganisms [13]. The use of probiotics is based on their ability to help to reestablish microbial–host balance in the digestive system after disruption of normal function by stress, infection or medical therapy [1]. The concept of prebiotics was introduced by Gibson and Roberfroid (1995) [14], as “a non-digestible food ingredient that beneficially affects the host by selectively stimulating the growth and/or activity of one or a limited number of bacteria in the colon and thus improves host health”. Unlike with probiotics, in which allochthonous microorganisms are introduced into the gut and need to compete against established colonic microbial communities, an advantage of using prebiotics to modify gut function is that the target bacteria are already commensal to the intestine. Consequently, prebiotics may be a more practical and efficient way to manipulate the gut microbiota than probiotics [15]. Mannan-oligosaccharides (MOSs) are components of *Saccharomyces cerevisiae* cell walls and may directly influence colonic bacterial populations by acting as an available substrate [16] or indirectly by influencing the immune system. In addition to directly influencing the population of beneficial bacteria within the colon, MOSs may also act indirectly by preventing binding of certain invading bacteria [17]. Numerous *Escherichia coli* strains and *Salmonella* species exhibit this mannan-binding behavior [18]. A supplemental MOS has been previously shown to affect the immune system of dogs [19,20]. However, if for any reason, such as disease, aging or antibiotic or drug therapy, the appropriate health-promoting species are not present in the gut, prebiotics are unlikely to be effective [21].

In the case of acute diarrhea, the structure of the intestinal barrier may be damaged and inflamed. Therefore, in addition to the modulation of the microbiota, it is helpful to use substances that facilitate in reparative processes and have anti-inflammatory effects. A natural compound with this quality is tannic acid, whose antidiarrheal properties can be attributed to a combination of several factors: it improves the properties of the epithelial barrier, inhibits the secretion of intestinal fluids and possesses antioxidant, astringent, antibacterial and anti-inflammatory capacities [22,23,24]. Zinc also has beneficial effects on the intestine in the case of acute diarrhea as it has a direct effect on intestinal villus, brush border disaccharidase activity and intestinal transport of water and electrolytes [22]. Moreover, it has been reported that the repair process of the intestinal wall can be assisted through the administration of nucleotides such as dietary purines and pyrimidines, serving as precursor units of DNA and RNA, especially under a relatively low intake of proteins. This could have an important role in intestinal development and maturation as precursors of nucleic acid in the salvage pathway, thus sparing the organism from the effects of their synthesis [23].

In this study, we compared the recovery of two different types of therapies on patients divided into two separate groups over the course of 6 days. One treatment was conventional drug therapy with the antimicrobial combination metronidazole–spiramycin, which is often used in clinical practice for uncomplicated diarrhea to combat the increase in pathogenic bacteria that could accompany acute diarrhea, regardless of the direct cause. Alternatively, we tested the effects of a nutraceutical complementary feed with galenic capsules composed of zinc oxide, chestnut extract, tannic acid, zinc, vitamin B12 and nucleotides, enriched with prebiotics and probiotics.

## 2. Materials and Methods

The study design is a prospective, randomized and single-blinded trial.

### 2.1. Animals

Thirty dogs with acute non-hemorrhagic diarrhea were enrolled in the study between December 2017 and March 2018. All dogs were patients of a first opinion veterinary practice (Veterinario Dr. Carlo Trevisan, Guardiagrele, CH, Italy).

Dogs without a history of previous gastrointestinal signs, with regular antiparasitic treatments and vaccinations, were eligible for this study.

Inclusion criteria for recruitment were acute non-hemorrhagic diarrhea with or without vomiting; 6 months of age or older; body weight ranging between 5 and 40 kg. Exclusion criteria were signs of systemic inflammation; sepsis (rectal temperature <37.0 °C or >39.0 °C; heart rate >140/min; depressed mental state; PCV > 58%, WBC < 5 × 10^9^/20 × 10^9^/L), band neutrophils >1.5 × 10^9^/L; clinical problems requiring hospitalization (for example: significant dehydration, signs of systemic disease); endoparasites; presence of diarrhea for more than 10 days; treatment with antibiotics in the last 30 days; treatment with anti-inflammatories or corticosteroids in the last 7 days. Dogs whose clinical status worsened during the study or had an increase in their Canine Acute Diarrhea Severity Index (CADS) score [24] by 4 points or more after the trial started were excluded from the study after first inclusion.

### 2.2. Study Design

Subjects were randomly (1:1) assigned to two groups (15 dogs per group) and all included dogs were fed with dry industrial food for adult dogs—Royal Canin Gastrointestinal^®^ (Royal Canin Italia, Milano, Italy) (Table S1) and were treated by the owners at home for 6 days as follows.

Group A was treated with a symbiotic in tablets composed of tannic acid and microencapsulated zinc oxide, chestnut extract, probiotics (*Enterococcus faecium* SF68 3.5 × 10^10^ CFU/g,), MOS, micronized dried carob flour, dicalcium phosphate, potassium citrate (potassium salts of organic acids), magnesium stearate, nucleotides and vitamin B12; daily oral administration of one tablet per 5 kg of body weight (Table 1). Group B received an antimicrobial combination of metronidazole (12.5 mg/kg) and spiramycin (7500 I.U./kg) once a day per os.

To evaluate the clinical course of the symptomatology during the 6 days of treatment, on the day of presentation and inclusion in the study, defined as day 1, all dogs were evaluated with the CADS Index (Table 2). Each owner compiled data on a daily basis in a diary that was provided (Figure S1) and, from day 1 to day 6, the score was reassessed by the owner and documented in the diary.

**Figure 1:**
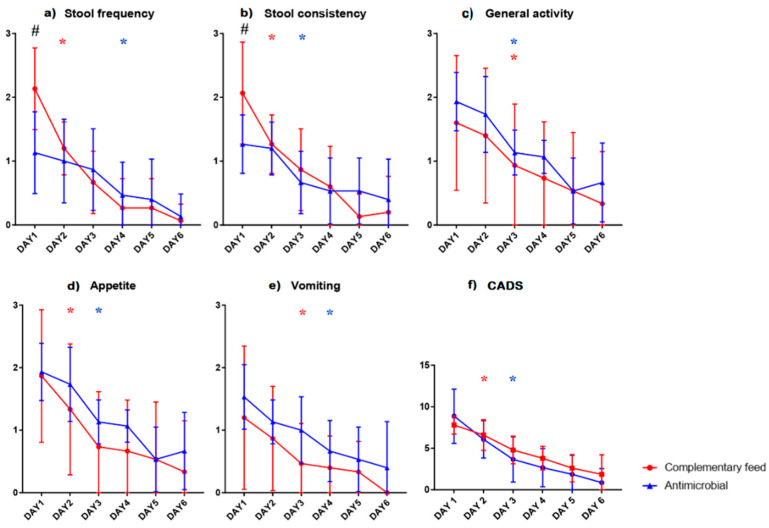
Mean values (y axes) for the parameters (**a**) stool frequency, (**b**) stool consistency, (**c**) general activity, (**d**) appetite, (**e**) vomiting and (**f**) CADS in the groups of dogs treated with complementary feed (group A; red) and antimicrobials (group B; blue). * Day on which the mean significantly decreased compared to day one for each group; color indicates group (*p* < 0.05). # Difference between treatments at any point in time (*p* < 0.05).

Owners collected the feces of their dogs on day 0, day 6 and day 30 in order to perform analysis of the intestinal microbiota. Feces were stored frozen at −20 °C until processing (Figure 1).

### 2.3. Clinical Procedures

The veterinarian collected the dogs’ history and performed the physical examination on day 0 and day 6, reporting the data in the patient medical record.

Complete blood count, biochemical examination and fecal examination (flotation test, Giardia antigen with IDEXX SNAP Giardia Test (DEXX SNAP^®^ Giardia, IDEXX Laboratories Inc., Westbrook, ME, USA) in order to detect possible parasitic infestation were performed on all dogs prior to the enrollment in the study.

### 2.4. Microbiome Analysis

DNA was extracted from each fecal sample (100 mg) using the MoBio Power soil DNA isolation kit (MoBio Laboratories, Carlsbad, CA, USA) according to the manufacturer’s instructions. Quantitative PCR assays (qPCR—BioRad Laboratories, CA, USA) were performed for key bacterial taxa included in the final canine qPCR-based DI which have all been shown to be altered in previous sequencing and qPCR-based studies between healthy dogs and dogs with intestinal inflammation (i.e., total bacteria, *Faecalibacterium*, *Turicibacter*, *Escherichia coli*, *Streptococcus*, *Blautia*, *Fusobacterium* and *Clostridium hiranonis*) as previously described [25,26,27]. While all of the bacterial taxa analyzed were significantly different between healthy dogs and dogs with CE, the combined results expressed as DI had the highest discriminatory power. This is explainable due to the known individuality in the abundances of specific bacteria taxa between dogs [28], and measurement of the abundance of one single taxon cannot distinguish between health and disease with high accuracy.

Briefly, qPCR reactions were performed using SYBR Green-based reaction mixtures. The qPCR results were expressed as the log amount of DNA for each bacterial taxon/10 ng of isolated total DNA. The results of the qPCR assays were also combined to calculate the Dysbiosis Index, which expresses the degree of dysbiosis as a single numeric value. A negative DI indicates eubiosis, whereas a positive DI indicates dysbiosis [25,26,27].

### 2.5. Statistical Analysis

All datasets were tested for normal distribution using D′Agostino and Pearson omnibus normality tests. The variations of the bacterial taxa, the CADS and the five parameters used to calculate it over time were analyzed using 2-way repeated measures ANOVA, followed with Dunn’s multiple comparison test. A *p* value < 0.05 was statistically significant for all analyses. All statistics were performed with GraphPad Prism 7.03 (GraphPad Software Inc., San Diego, CA, USA).

## 3. Results

Demographics and baseline characteristics of the 30 dogs, meeting all inclusion criteria and completing the study according to protocol guidelines, are summarized in Table 3. Complete blood count and serum chemistry were within normal limits and fecal examinations were negative in all included dogs. During the clinical trial, no adverse effects were recorded in enrolled dogs.

All dogs showed a small intestine involvement. Stool consistency and stool frequency were higher at presentation (day 1) in the dogs treated with complementary feed (group A) compared to the antimicrobial group (group B) (*p* = 0.0026). Stool frequency and consistency significantly decreased on day 2 in group A compared to baseline and both parameters decreased significantly compared to baseline on days 3 and 4 in the dogs of group B (Figure 1). However, appetite and vomiting in group A had a significantly lower score one day earlier, when compared to baseline, than group B (Figure 1).

No differences were observed for the composite CADS Index on day 1. Significant reduction in the CADS Index was achieved 1 day faster in group A than in group B. Overall, a progressive reduction, over time, in the CADS Index was observed in dogs belonging to both study groups.

The microbiome analysis showed a similar pattern in study dogs. In particular, no significant differences were observed between group A and B for any of the analyzed bacterial taxa at baseline. *Faecalibacterium*, *Turicibacter*, *Escherichia coli*, *Streptococcus*, *Blautia*, *Fusobacterium* and *Clostridium hiranonis* followed the same trend over the time, regardless of the product received.

**Figure 2:**
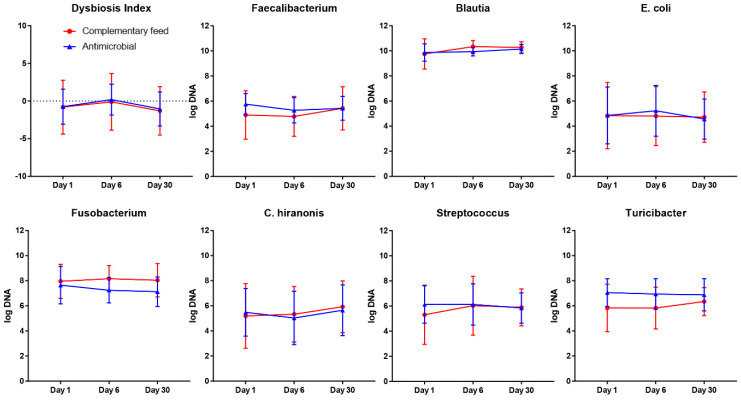
Bacterial taxa and the Dysbiosis Index in the groups of dogs treated with complementary feed (group A; red) and antimicrobials (group B; blue). There were no significant differences at any point in time between groups.

Similarly, the Dysbiosis Index did not significantly differ between the two study groups at baseline or any other time points (Figure 2).

## 4. Discussion

In this study, we compared the effect of a nutraceutical, with a commonly used antimicrobial combination, on the progression of acute diarrhea in dogs. The data obtained from the daily questionnaires indicated that the administration of both treatments improved the clinical conditions in every patient. In the study groups, no differences were observed in the CADS Index on day 1, and this score never decreased over the course of the clinical trial in either group. The difference between groups on day 1 regarding stool frequency and consistency could influence the result, as group A had a greater range of improvement. It is worth noting that the dogs receiving the nutraceutical feed showed a marked improvement of appetite, stool consistency and frequency as early as day 2. This suggests a benefit of nutraceutical therapy in the treatment of acute diarrhea, because a 1-day decrease in clinical signs would be appealing for clients challenged with managing a diarrheic pet.

A second aim of this study was to evaluate the effects of both treatments on the intestinal microbiota. This was done by measuring abundances of main bacterial taxa, which previous studies revealed to have a significant alteration in dogs with intestinal disease [25,27,29,30]. Interestingly, a high standard deviation was observed, suggesting the presence of a high variability both within and among individuals and throughout the study period, mirroring the natural inter- and intra-individual microbiome variability over time.

It is hypothesized that antimicrobial treatment may lead to an increase in the Dysbiosis Index, an increase in *E. coli* and a depletion of beneficial anaerobic bacterial groups (e.g., *Faecalibacterium*, *Blautia*, *Turicibacter*) as observed previously for treatments with metronidazole in healthy dogs. Surprisingly, at no point in time were there any differences in any of the evaluated bacterial taxa or the Dysbiosis Index between treatments. This contrasts with the abovementioned studies, which showed a clear increase in the Dysbiosis Index and *E. coli*, and decreases in *Faecalibacterium*, *Blautia and Turicibacter* after administration of metronidazole (12.5 mg/kg and 15 mg/kg BID) as monotherapy [29,30].

It is unclear why the combination therapy of metronidazole and spiramycin, as used in this study, did not induce similar changes in the fecal microbiota, despite using the same qPCR assay as before [29]. The administered antimicrobial was given only once a day in this study, in contrast to the previous studies, and this may be one reason for the lack of induction of dysbiosis. Since no full 16S rRNA gene analysis was performed in this study, it is possible that we missed some bacterial taxa that may have been affected by the antimicrobial.

Recently, a revolutionary detection method was implemented to obtain quantitative insight at a deep phylogenetic resolution by combining 16S-targeted Illumina sequencing with flow cytometry [31]. Another study used this method, attesting that yeast-based ingredients, used as prebiotics, have a large potential to beneficially stimulate the microbiota activity and composition of cats and dogs, thus suppressing outgrowth of opportunistic pathogens, such as *E. coli* [32].

However, as mentioned above, the assay used here has previously been shown to reveal changes in microbiota in canine diarrhea as well as due to antimicrobial therapy. Although no obvious difference in effects were observed on the intestinal microbiota between nutraceutical and metronidazole–spiramycin treatment, antimicrobial therapy should be reserved only for dogs with evidence of infectious causes and in those cases showing signs of systemic inflammatory response syndrome. Indeed, the global concern for rising antibiotic resistance associated with indiscriminate use of antimicrobials suggests the necessity of avoiding empirical and injudicious use of these molecules in diarrheic dogs. Antibiotic resistance represents one of the most serious and imminent health-related problems worldwide (WHO 2017). Moreover, it has been observed that antibiotic-associated gastrointestinal side effects (AAGSs) occur in 5% to 39% of people, with higher incidences of up to 70% in children [33]. Although the incidence of AAGSs in dogs and cats is largely unknown, prolonged derangements in the microbiome have been demonstrated in animals receiving other antibiotics, such as tylosin [34].

Acute diarrhea, with no additional known health condition(s), is common in dogs and is often considered to be related to diet or stress—although bacterial, viral or protozoal infections may also be involved. A balanced microbial environment prevents invasion by pathogens, influences the gut structure, provides nutritional benefits to the host (e.g., production of SCFAs, aids in the synthesis of B and K vitamins) and strengthens the immune system by interacting with key immunomodulatory cells [35]. SCFAs have many further effects on the colonic mucosa: they affect the pH of compartments in the mucosa, cell swelling and stimulation of mucin release and of mucosal blood flow [36]. Dogs with acute diarrhea had significantly different microbial communities and lower species richness compared to healthy dogs [11]. In addition to the microbiota, the integrity of the intestinal barrier is also essential for host health, as the intestinal epithelium has the complex task of providing a barrier and allowing nutrient and water absorption. Therefore, an intact intestinal barrier is critical to normal physiological function and prevention of disease [37,38]. The combined use of zinc, tannins, nucleotides, probiotics and prebiotics as nutraceuticals may have a synergistic affect in the restoration of an altered intestinal barrier and microbiota wellness, to promote the immune response and the management of diarrheal symptoms.

All dogs received a new diet providing hydrolyzed proteins, fructo-oligosaccharides (FOSs), MOSs, antioxidants such as zinc, selenium, manganese and omega-3 and omega 6-fatty acids. Therefore, the clinical response and the changes observed during the study could be due to the diet, rather than the use of the symbiotic or the antibiotic combination. Moreover, it is not clear to what extent each active ingredient of symbiotic product contributed to the positive effect identified here and further studies are warranted in order to elucidate the role of each ingredient. Several mechanisms can be proposed, such as the inhibition of the growth of the pathogens, the modulation of the gastrointestinal immune function, the modulation of the gastrointestinal microbiome or the modulation of gastrointestinal motility, the connection of water and toxins or a combination of these factors.

A limitation of this study was that it did not include a group receiving placebo treatment. Therefore, it is unknown whether the same parameters would have also improved in dogs with acute diarrhea without any specific treatment. This was due to the common perception by practicing veterinarians that antimicrobial therapy leads to faster resolution of clinical signs, although no studies exist that would validate such perceptions. In fact, a survey assessing common treatment of acute diarrhea in general practice has revealed that 71% of veterinarians in the United Kingdom used antimicrobials as first line treatment [4]. Studies involving use of probiotics on dogs with acute diarrhea showed a faster resolution of symptoms in the treated group compared to placebo [3,27,39]. Recently, studies have suggested that there is no clear benefit of antimicrobial therapy in dogs with non-hemorrhagic diarrhea [40] or hemorrhagic diarrhea in dogs without signs of sepsis [30]. Based on these studies, inclusion of a placebo group in future studies is warranted, as the absence of a control group represents a limitation for the current study, considering that acute diarrhea is often self-limiting.

## 5. Conclusions

This trial highlighted how a treatment based on plant extracts, minerals, vitamins, probiotics and prebiotics can have similar clinical effects compared to an antibiotic formulation. Treatment with this nutraceutical product may represent a valid alternative to commonly used antimicrobial therapy as it was associated with faster clinical recovery. Further studies are needed to confirm the observations made in the present trial.

## Figures and Tables

**Table 1:** Composition of nutraceutical complimentary feed (tablets 1400 mg).

Active Ingredient	Unit	Quantity
ZT455C ^1^	mg/tab	500
Mannanoligosaccharides	mg/tab	300
Carob flour	mg/tab	140
Nucleotides	mg/tab	50
Enterococcus faecium (35 billion CFU/g) DSM 10663/NCIMB 10415	mg/tab	40
Vitamin B12 (0.1%)	mg/tab	5
**Excipients**	**Unit**	**Quantity**
Dicalcium phosphate	mg/tab	190
Vegetable appetizing	mg/tab	75
Microcrystalline cellulose	mg/tab	40
Potassium citrate	mg/tab	30
Silicon dioxide	mg/tab	15
Magnesium salts of fatty acids	mg/tab	15

^1^ ZT455C: combination of zinc oxide plus tannic acid microencapsulated.

**Table 2:** Scoring system of the Canine Acute Diarrhea Severity Index.

Parameter	0	1	2	3
Activity	Normal	Mild	Moderate	Severely decreased
Appetite	Normal	Mild	Moderate	Severely decreased
Vomiting	Normal	1 ×/d	2–3 ×/d	> 3 ×/d
Stool consistency	FeSc ^1^ 2–3/7	FeSc 4–5/7	FeSc 6/7	FeSc 7/7
Stool frequency	Normal	3 ×/d	4–5 ×/d	> 5 ×/d
Activity	Normal	Mild	Moderate	Severely decreased

^1^ FeSc: fecal scoring system according to the Bristol Stool Chart.

**Table 3:** Characteristics of the 30 dogs participating in the clinical trial. All characteristics reflect baseline values immediately before study enrollment.

Variable	Group A	Group B	*p*-Value
Age (years)	6.2 ± 3	7.9 ± 3.3	0.13
Sex (male/female)	10/5	9/6	0.57
Weight (kg)	20.4 ± 10.8	17.8 ± 13.1	0.30

## Data Availability

The data presented in this study are available on request from the corresponding authors. The data are not publicly available due to privacy protection.

## Supplementary Materials

Available online at https://www.mdpi.com/article/10.3390/ani11061484/s1. Table S1: Composition of dry food Royal Canin Gastrointestinal^®^, Figure S1: Daily diary filled in at home by the owner.
