# Supplementary material for: Comparison of the Therapeutic Effect of Treatment with Antibiotics or Nutraceuticals on Clinical Activity and the Fecal Microbiome of Dogs with Acute Diarrhea
Source: Animals (Basel). 2021 May 21;11(6):1484. doi: 10.3390/ani11061484 (PMC8223982; doi:10.3390/ani11061484)
Supplement: Supplementary file 1 [file animals-11-01484-s001.zip › animals-1217124-supplementary.pdf]

Supplementary material

Comparison of the therapeutic effect of treatment with antibiotics or nutraceuticals on clinical activity and the fecal microbiome of dogs with acute diarrhea

Giulia Pignataro[1*], Roberta Di Prinzio[1], Paolo E. Crisi[1*], Benedetta Belà[1], Isa Fusaro[1], Carlo Trevisan[2], Luigi De Acetis[3] and Alessandro Gramenzi[1]

[1] Faculty of Veterinary Medicine, University of Teramo, Strada Provinciale 18, 64100 (TE), Italy;
[2] Veterinary practice, "Ambulatorio Veterinario Dr. Carlo Trevisan", Guardiagrele, Via Occidentale 91, 66016 (CH), Italy;
[3] Veterinary practice, "Ambulatorio Veterinario Dr. Luigi De Acetis", Caramanico Terme, (PE), Via Santa Croce, 65023, Italy;
* Correspondence: gpignataro@unite.it (G.P.); pecrisi@unite.it (P.E.C.)

Supplementary Table S1. Composition of dry food Royal Canin Gastrointestinal®.

Composition		
Rice		
Dehydrated poultry protein		
Maize		
Animal fats		
Hydrolyzed animal proteins		
Egg powder		
Yeasts products		
Beet pulp		
Soya oil		
Fish oil		
Vegetables fibers		
Minerals		
Psyllium		
Husk and seeds		
Hydrolyzed yeasts (0.2%)		
Marigold extract		
FOS (0.5%)		
**Nutritional additives**	**Unit**	**Quantity**
Vitamin A	IU/kg	16500
Vitamin D3	IU/kg	1000
Iron	mg/kg	41
Iodine	mg/kg	4.1
Copper	mg/kg	12
Manganese	mg/kg	53
Zinc	mg/kg	129
Selenium	mg/kg	0.07
**Analytical constituents**	**Unit**	**Quantity**
Protein	%	25.0
Fat	%	20.0
Crude ash	%	6.6
Crude fibers	%	1.9
EPA/DHA	%	0.31
Omega-3 fatty acids	%	0.85
Omega-6 fatty acids	%	4.28
Sodium	%	0.43
Potassium	%	0.65

FOS: fructo-oligosaccharides; EPA: eicosapentaenoic acid; DHA: docosahexaenoic acid.

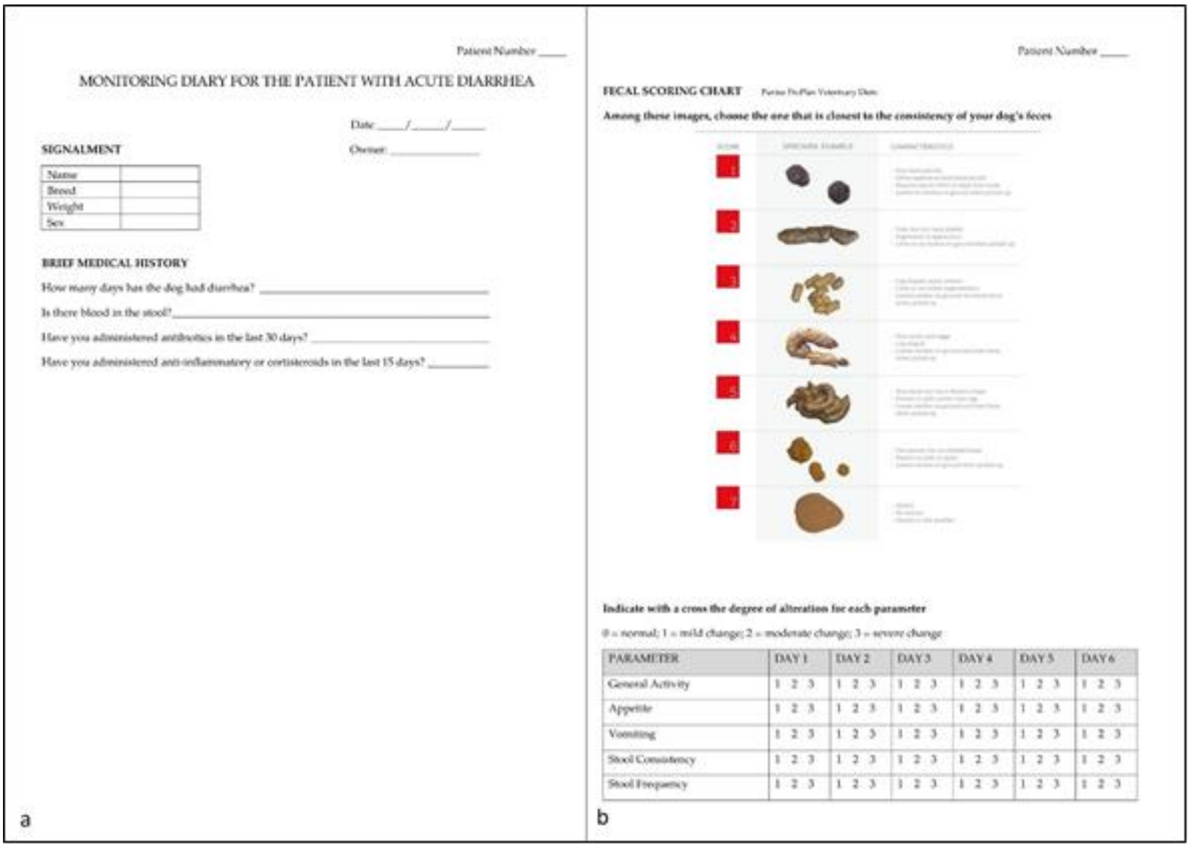

Patient Number ____

MONITORING DIARY FOR THE PATIENT WITH ACUTE DIARRHEA

Date ____/____/____

Owner: ____________

SIGNALMENT

Name	
Breed	
Weight	
Sex	

BRIEF MEDICAL HISTORY

How many days has the dog had diarrhea? ____________________

Is there blood in the stool? ____________________

Have you administered antibiotics in the last 30 days? ____________________

Have you administered anti-inflammatory or cortisteroids in the last 15 days? __________

a

Patient Number ____

FECAL SCORING CHART Purina ProPlan Veterinary Diets

Among these images, choose the one that is closest to the consistency of your dog's feces

1

2

3

4

5

6

7

Indicate with a cross the degree of alteration for each parameter

0 = normal; 1 = mild change; 2 = moderate change; 3 = severe change

PARAMETER	DAY 1	DAY 2	DAY 3	DAY 4	DAY 5	DAY 6
General Activity	1 2 3	1 2 3	1 2 3	1 2 3	1 2 3	1 2 3
Appetite	1 2 3	1 2 3	1 2 3	1 2 3	1 2 3	1 2 3
Vomiting	1 2 3	1 2 3	1 2 3	1 2 3	1 2 3	1 2 3
Stool Consistency	1 2 3	1 2 3	1 2 3	1 2 3	1 2 3	1 2 3
Stool Frequency	1 2 3	1 2 3	1 2 3	1 2 3	1 2 3	1 2 3

b

Supplementary Figure S1. Daily diary to fill at home by the owner: first page (a) and second page (b).
